# A longitudinal analysis of the fluctuation of food stores in Belo Horizonte, Minas Gerais, Brazil

**DOI:** 10.1186/s12889-023-17350-8

**Published:** 2023-12-07

**Authors:** Patrícia Pinheiro de Freitas, Mariana Souza Lopes, Bruna Vieira de Lima Costa, Denise Marques Sales, Mariana Carvalho de Menezes, Patrícia Constante Jaime, Aline Cristine Souza Lopes

**Affiliations:** 1 Research Group on Nutrition Interventions, Universidade Federal de Minas Gerais, Av. Alfredo Balena, 190, room 316, Santa Efigênia, Belo Horizonte, MG 30130-100 Brazil; 2 Nutrition Department, Universidade Federal de Minas Gerais, Av. Alfredo Balena, 190, room 314, Santa Efigênia, Belo Horizonte, MG 30130-100 Brazil; 3 Institute of Geosciences in Geography (IGC), Universidade Federal de Minas Gerais, Av. Antônio Carlos, 6.627, Pampulha, Belo Horizonte, MG 31270-901 Brazil; 4 School of Nutrition, Research Group on Nutrition and Collective Health, Universidade Federal de Ouro Preto, St Dois, 607, room 64, Ouro Preto, MG 35400-000 Brazil; 5 Department of Nutrition, School of Public Health, University of São Paulo, Av. Dr Arnaldo 715, São Paulo, SP 01246-904 Brazil; 6 Department of Nutrition, Universidade Federal da Paraíba, Campos I, Cidade Universitária, Castelo Branco, João Pessoa, 58051-900 Brazil

**Keywords:** Food environment, Fruits and vegetables, Longitudinal study, Food stores, Health service environments

## Abstract

**Background:**

Changes in food environments have the potential to affect consumption, nutritional status, and health, and understanding these changes is of utmost importance. This study, therefore, aimed to examine the fluctuation of food stores that sell fruits and vegetables over five years in the health promotion service area of Primary Health Care (PHC) in Belo Horizonte, Minas Gerais, Brazil.

**Methods:**

This was an ecological study that used data from a food environment audit conducted in the realm of Brazilian PHC. Buffers of 1 mile (equivalent to 1600 m) were created around health promotion services to define food environments. All food stores and open-air food markets that sold fruits and vegetables (FV) within this buffer area were considered eligible. The data collection was performed during two periods: the baseline, in 2013, and after five years, in 2018. This study compares the fluctuation by the type of stores and according to the health vulnerability index (HVI).

**Results:**

After 5 years, 35.2% of the stores were stable; 154 stores were closed, and 155 were opened. The stability was greater in low-vulnerability areas, and the fluctuation differed by type of store only for areas with high vulnerability. The number of supermarket decreased in high HVI territories; and local stores, showed greater stability when compared to specialized FV markets.

**Conclusions:**

The differences in store fluctuations according to the vulnerability of areas demonstrate the importance of food supply policies considering the local characteristics to reduce inequities of access to healthy foods.

**Supplementary Information:**

The online version contains supplementary material available at 10.1186/s12889-023-17350-8.

## Background

The food environment has the potential to influence food purchases, dietary behaviors, as well as health outcomes [1]. Exposure to a limited food environment may amplify individual and community risk factors associated with poor diets. By contrast, great accessibility to stores that sell healthy food, like fruits and vegetables (FV), contributes to healthy diets and promotes health [2].

In Brazil, studies on the role of different food stores in the purchase profile are divergent. In general, supermarkets are the main food purchasing establishments, followed by small stores. The supermarkets it is the place where 60.4% of the energy from ultra-processed foods. On the other hand, the purchase pattern characterized by use of street fairs, local stores and small specialized stores was associated with a smaller consumption of ultra-processed products [3]. Furthermore, specialized FV stores make healthy eating easier [4].

Changes in the distribution of food stores within an area are common and may reflect changes in consumer behavior, sales dynamics, and neighborhood development. On the other hand, when the stores that close have the same characteristics as the stores that open, there is a certain stability in the environment. However, if the stores that close have different characteristics from the stores that open, there may be a change in the dynamic of local retail. The closing of stores may require the adaptation of consumers to purchase food, especially if it represents only access to a particular food group [5].

Changes in the food environment need to be considered in studies on nutrition and health. In scenarios where there is no recent data about the food environment, the access to food stores may be unreal if stability is presumed when the change occurs. The results could be spurious findings and false conclusions about the association between the food environment and health. Furthermore, the characterization of changes in the food environment should guide the development of intersectoral public policies for health promotion; and the monitoring must be constant in order to observe changes and make necessary adjustments. If changes have not been evaluated, there may be less allocation of resources to areas that need it [6].

The analysis of changes in food environments is complex because most studies are cross-sectional and use secondary databases from commercial cooperatives, public governmental, telephone, and/or internet directories. Since updating data is time-consuming, the available data is often outdated and unreliable. In addition, the accuracy of this type of information is controversial, especially for regions with worse socioeconomic conditions [6–8]. Therefore, this type of data should be used with caution, especially when the food environments are dynamic [9]. In this sense, inspecting the food environment, especially in dynamic places, becomes essential to explore the food environment in different dimensions, to build healthy environment [7].

The existence of spaces that encourage physical exercise and healthy eating are examples of initiatives to build healthy environments. In this sense, the Unified Health System (SUS, in Portuguese) proposed such as the Health Academy Program, (*Programa Academia da Saúde, PAS, in Portuguese)*, spaces containing infrastructure, equipment, and human resources to offer health promotion actions, mainly physical activity and healthy eating, at no cost to interested people. More than 2,900 Brazilian municipalities have PAS units, People can use the PAS by spontaneous demand or when they are sent by professionals of other public healthcare services. The PAS users are mostly women, with a low level of education and income, who have a high prevalence of chronic non-communicable diseases (NCDs) and overweight [10].

The integration with exercise and healthy food environment in the same place, like PAS, can potentiate their effect on health. Considering these aspects, this study aimed to examine the fluctuation of stores that sell fruits and vegetables over five years in the Health Promotion Service area.

## Methods

### Setting and study design

This ecological study was conducted in Belo Horizonte, the capital city of Minas Gerais, Brazil. The city is the sixth most populous in the country and the eighth on the South American continent, with an estimated population that exceeds 2.3 million inhabitants in 2022 [11]. Belo Horizonte is divided into administrative regions where all health policies and actions are planned and administrated.

PAS units was the setting of this study and has been a health policy in Belo Horizonte since 2006. The routine activities of PAS in Belo Horizonte are physical exercise, guided by a physical educator, for 60 min, three times a week. The users also participate in health education activities developed by health professionals according to demands in the region [12].

PAS users, in general, lives next to the service, because Primary Health Care (PHC) in the city are territorialized. In the face of this characteristics and because is a key component of the promotion of healthy behaviors and environment, the PAS was chosen as the setting of this study.

### Study sample

The study sample was calculated considering the total number of PAS units in the city. The inclusion criteria of the units were: must be open in the morning and be located in an area of medium and high vulnerability to health (based on the Health Vulnerability Index, which is explained below), as it constitutes the predominant period and area of PAS operation in the municipality; it was not the subject of research related to food and nutrition in the last 24 months, and it has been operating since 2012 (period of the sampling process) [13].

From 50 units operating at the time of the sampling process, 42 were found to be eligible and eighteen units were randomly selected for the study via stratified sampling by nine administrative districts of the city, with two units per region. These units were representative of PAS units in medium and high health vulnerability in Belo Horizonte, with a 95% confidence interval (CI) and < 1.4% error. More information about methods and sampling can be seen in a previous publication [13].

To characterize the food environment, stores that sell FV and open-air food markets within the buffer regions, with a radius of 1 mile (1600 m) around each selected unit of the PAS. This radius was chosen because it is a usual walkable distance between adults (95%), and is frequently used in other studies [14, 15; 16]. In addition, our study evaluated only stores selling FV to further the studies on these food groups according to the capacity they have to promote health [14].

### Data collection

The data collection was performed through an audit in the eligible food stores in two periods: the baseline, in 2013, and after five years, in 2018. The research team consisted of dieticians and undergraduate students from the Department of Nutrition. They performed the audit in pairs, supervised by a researcher. All interviewers were previously trained based on the field manual and the review of this training was conducted before starting each period of data collection.

At baseline, the first step to identify the eligible stores and open-air food markets was to check their registrations at City Hall. The name and address of the store contained in the buffers were selected to guide the interviewers in the audit. If the store in the public database was not found in the audit, contact by telephone or search using Google Street View was used. In addition, non-registered food stores found randomly in the region during store audits were also included, allowing us to update the database [7].

Data collection included the registration of the stores (commercial name and record number at the National Register of Legal Entities - NRLE), obtained by interviews with the owners or employees, as well as addresses collected by the interviewer at the time of the audit. Through direct observation, the interviewers checked the sale of FV to select included stores. More information about baseline data collection can be seen in a previous publication [7].

In 2018, after 5 years of the baseline, a new audit was conducted, beginning from the list of names and addresses of stores from the data collected in 2013. New stores found in this audit were also included. The collected information and the procedures were similar to the baseline.

### Food store fluctuations

The differences in the total number of food stores between 2013 and 2018 were used to analyze food store fluctuations. To determine changes over the five years, the following information was used: (1) store commercial name; (2) record number (NRLE), and (3) address. If there was a minor difference in a store name, the 2018 NRLEs were compared to the 2013 NRLEs to determine if the store had transferred ownership. If the NRLEs were different, they were considered different stores, but if they were equal, they were considered the same store. The record number was used as a reference to determine the stability of the store, since although the location address is the same, the commercial purpose of the food may have changed.

Three categories were created to define the fluctuations in food stores based on the proposal by Folimena et al. [5]: (1) stable, which exist in both years; (2) closed, which existed in 2013 but not in 2018; (3) new stores, which were found in 2018 but did not exist in 2013. Closed food stores were given a sub-code to indicate if they were: (a) replaced by a different store that also sells FV; (b) stores not found, did not exist, or were occupied by another retail food type but did not sell FV. The definitions for each of these categories are described below (Table 1).

**Table 1:** Definitions of food store fluctuations^1^

Category	Description
Stable stores	Stores that remained open after five years (same name, address, and NRLE) and that participated or refused the audit.
Closed stores	Stores in the 2013 database that were not in the 2018 database or stores that no longer sell FV in 2018: *a) Replaced stores*: stores in the 2013 database replaced by a new establishment that sells FV in the 2018 database (new name and NRLE but same address). b) *Not replaced stores*: stores in 2013 database were not found or did not exist during the 2018 audit.
New stores	Stores in the 2018 database that did not exist in 2013 (comparing name and address).

*Note*: NRLE = National Register of Legal Entities; FV = Fruit and Vegetable. ^1^Source: adapted from Filomena, 2013

### Type of food stores

The food stores were classified into the following categories, adapted from a Brazilian study [15]: large chain supermarkets and grocery stores, specialized FV markets/stores and open-air food markets, local grocery stores, and delis and convenience stores. From this classification, three categories were proposed: (1) supermarkets; (2) specialized FV markets (specialized stores or open-air food markets); (3) local stores (local grocery stores, convenience stores, delis, and bakeries).

### Health vulnerability index

The Health Vulnerability Index (HVI) was developed by the Municipal Health Secretary of Belo Horizonte to guide the planning of health actions, including the PAS implementation site.

The HVI combines socio-economic and sanitation variables using the scale of analysis of the census sector obtained in the last Brazilian demographic census available until data collection. The variables were selected based on their discriminatory power of spatial inequalities; and are divided into indicators, as presented in Supplementary Material 1. HVI is categorized into four classes: low, medium, high, and very high risk, to point out priority areas for intervention and a more effective resource allocation. In areas with high HVI, the population presents an increased risk of negative health outcomes; and in areas with a medium HVI, the population’s health risk is similar to the municipality’s average. By contrast, the population in the regions with low HVI presents a low risk of negative health outcomes related to socioeconomic and sanitary conditions. Considering their similarities, in this study, the high and very-high-risk classes were grouped.

**Fig. 1:**
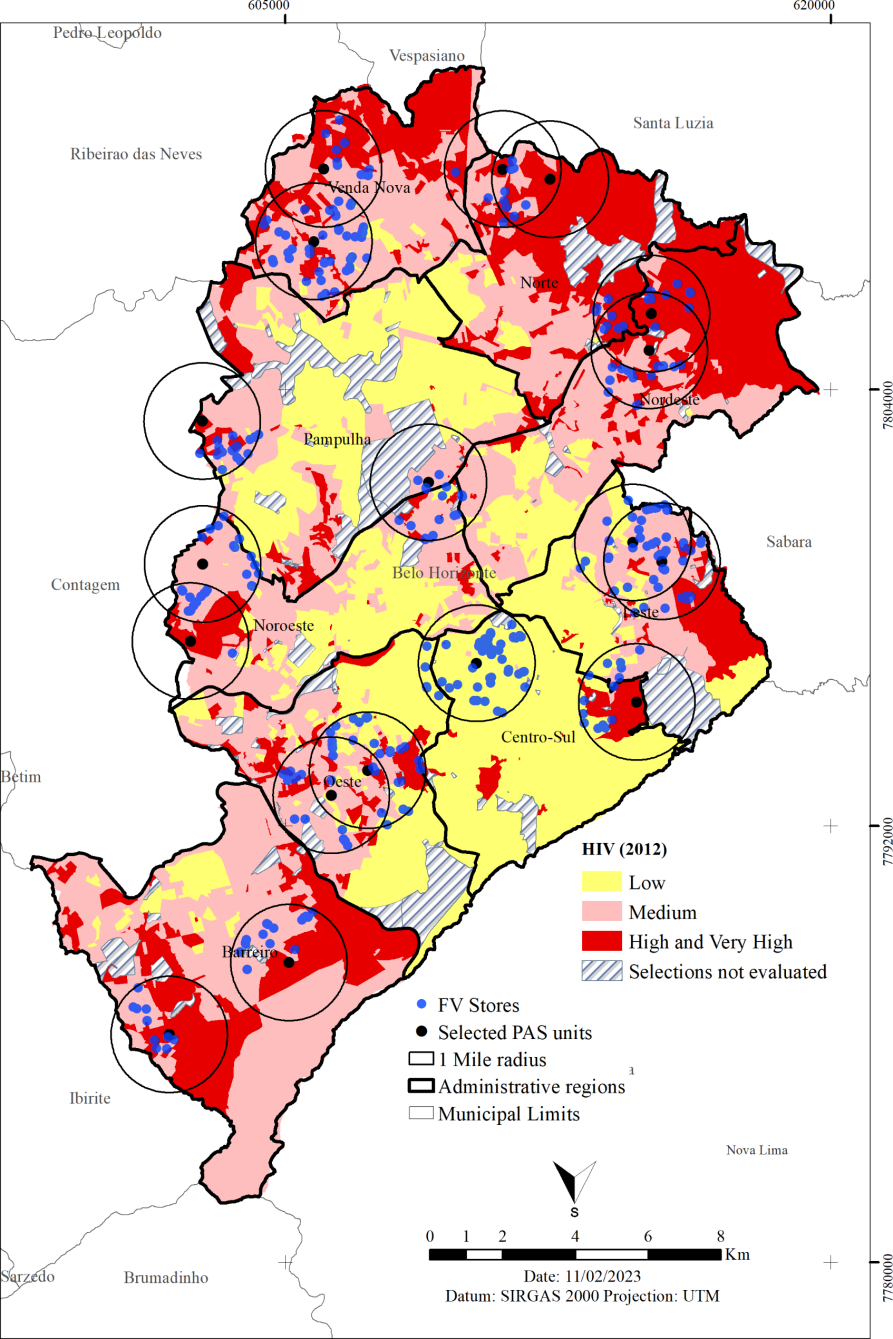
Location map of health promotion service, food stores, and vulnerability, Belo Horizonte, Brazil. *Note*: PAS = *Programa Academia da Saúde*. HVI = Health Vulnerability Index

The HVI of the area of the food store was defined from the address of the store (Fig. 1). The HVI data were obtained only in the first year of collection. In this way, it was possible to compare the same region in the two moments of analysis.

### Data analysis

The organization and spatialization of data were performed using ArcGis 10.3. Data were analyzed using Stata version 14, and p < 0.05 was considered significant.

The food stores that were present in a radius of more than one unit of the PAS were duplicated in the database to represent the food environment of each of the regions. Stores localized in the area without HVI information data were excluded. Thematic maps were elaborated to identify the location of the PAS units to define and specialize the study radius and to provide an overlap with the municipality’s HVI [16] layer to identify the different vulnerabilities of the selected areas. Moreover, through data spatialization, it was possible to identify the changes in store locations in 2013 and 2018. In another map, store data was overlaid with the population density obtained by using information from the 2010 demographic census aggregated to the human development unit (HDU).

The number of stores was presented by year, type of store, and HVI. Absolute number, frequency, and 95% CI of store fluctuation each year were presented according to the type of store by HVI. The variation in the number of food outlets among the analysis’s periods (absolute and percentage) and between the final (2018) and initial (2013) periods was quantified. Generalized linear models were used to analyze the trend in the number of food outlets. The chi-square test was used to compare the presence of stores in each year and to compare the store fluctuations and types by HVI.

To assess whether possible changes in the food environment occurred differently according to the proximity of the stores to the PAS units, the number of stores was described considering buffers of 1 mile around the PAS unit. The radius of 1 mile was maintained due to its previously mentioned relevance.

This study does not involve human data. However, those responsible for the stores authorized the audit and signed a written informed consent form. Considering the other objectives of the larger research that this study is part of, the protocol was approved by the Ethics Committee University (ETIC 0339.0.203.000–09) and the City Hall (0339.0.203.000-09 A).

## Results

**Fig. 2:**
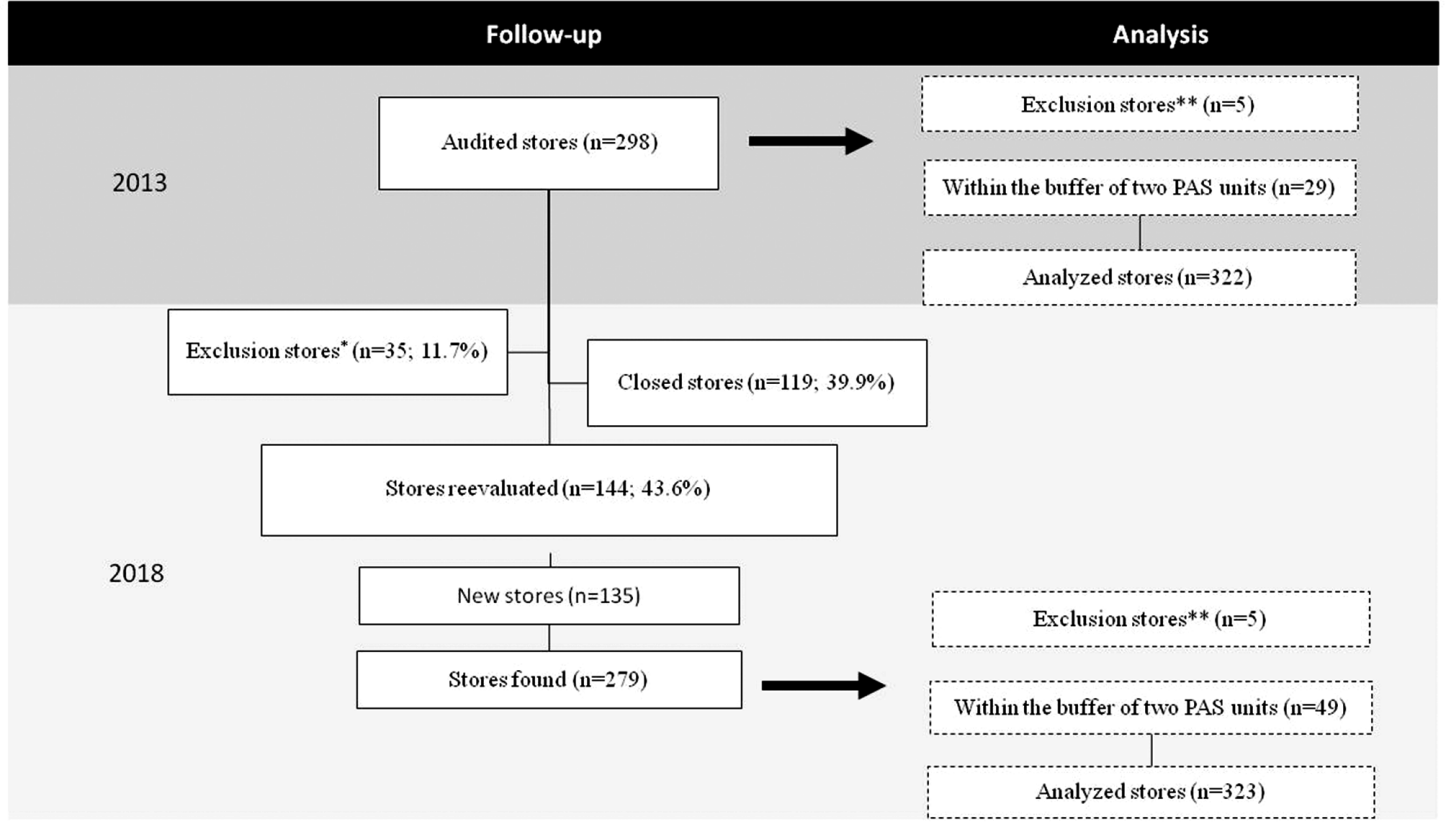
Food environment data after five years. Belo Horizonte, Minas Gerais, Brazil, 2013–2018. *Note*: PAS - Programa Academia da Saúde. ^*^Exclusion stores: n = 26, duplicity in the database; n = 8, no longer sold fruit and vegetables; n = 1, place could not be visited due to intense violence. ^**^Exclusion stores: not have HVI information

In 2013, 298 stores were audited. Of this total 39.9% were closed in 2018 and 4.7% refused to participate in the survey. In addition, 3.0% of the stores were excluded as they no longer sold FV, these stores were duplicated in the database, or they were located in an area of intense violence, which made data collection impossible. Another 135 new stores were found, totaling 265 stores inspected in 2018. After excluding the stores that did not have HVI (2013 = 5; 2018 = 5) and duplicating the stores that were in the buffer of two PAS units in the database (2013 = 29; 2018 = 46), 322 stores were analyzed in the baseline (2013) and 306 in the reevaluation after 5 years (2018) (Fig. 2).

The number of stores that sold FV did not change from 2013 to 2018 even when analyzed by type of store (Table 2).

**Table 2:** Number of food stores according to territorial vulnerability Belo Horizonte, Minas Gerais, Brazil, 2013–2018

	2013	2018	∆	p-value^a^
Total				
Supermarket	53	54	**1**	0.923
Specialized FV markets	207	206	**-1**	0.961
Local stores	62	63	**1**	0.929
Total	322	323	**1**	0.969
**Low HVI**				
Supermarket	16	22	**6**	0.332
Specialized FV markets	57	67	**10**	0.370
Local stores	7	9	**2**	0.618
Total	80	98	**13**	0.178
**Medium HVI**				
Supermarket	23	25	**2**	0.773
Specialized FV markets	111	103	**-8**	0.585
Local stores	37	37	**0**	1.000
Total	171	165	**-6**	0.743
**High HVI**				
Supermarket	14	7	**-7**	0.134
Specialized FV markets	39	36	**-3**	0.729
Local stores	18	17	**-1**	0.866
Total	71	60	**-11**	0.337

*Note*: HVI = Health Vulnerability Index Δ = stores in 2018 – stores in 2013

^a^Generalized linear models

**Fig. 3:**
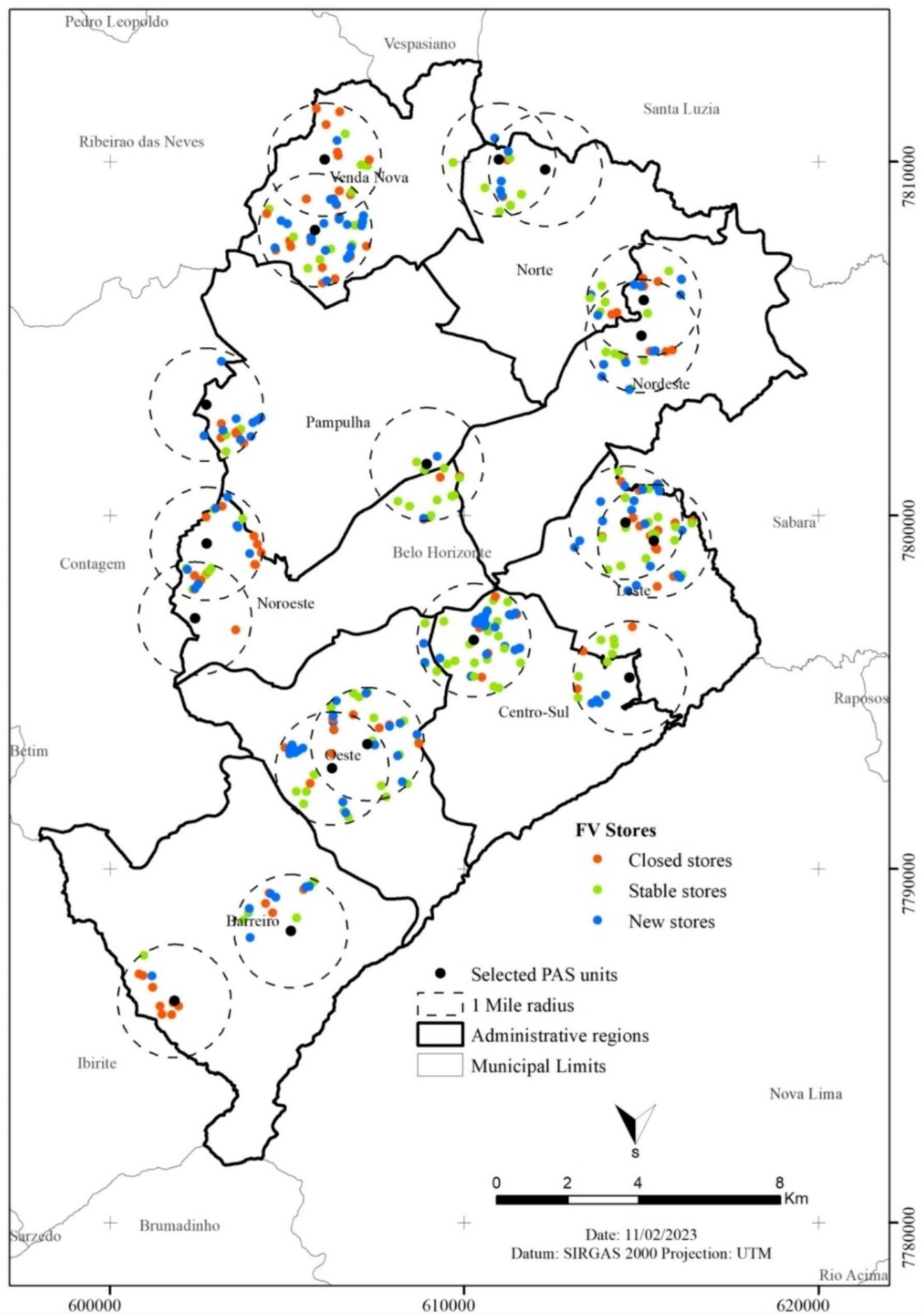
Location of the food stores and fluctuation over five years. Belo Horizonte, Brazil, 2013–2018. *Note*: Stables stores = store that remained open after five-years and participated or refused the audit; Closed stores = Stores in the 2013 database that were not in the 2018 database or stores that no longer sell FV in 2018. New stores = Stores in the 2018 database did not exist in 2013 (comparing name and address)

After 5 years, the stability of 35.2% of the stores was observed. In this period, 154 stores were closed, and 155 were opened. The total number of closed and open stores was very close, with the majority of closed stores not being replaced (92.2%) (Table 2). However, it is possible to observe a large number of closed stores in the administrative region of Barreiro. On the other hand, Pampulha, Venda Nova, Centro-Sul, and Leste had the highest number of new stores (Fig. 3).

The stability of the food environment was higher in the low HVI area (low = 45.9% vs. medium = 31.2% vs. high = 32.3%). Despite the increased stability, in the low HVI area, the highest proportion of new stores (low = 34.3% vs. medium = 33.2% vs. high = 28.3%) was observed. On the other hand, the high HVI area showed the highest proportion of closed stores (high = 39.4% vs. medium = 35.5 vs. low = 19.7%) (Table 3).

The fluctuation of the community’s food environment differed by type of store only in areas with high HVI, where local stores showed greater stability when compared to specialized FV markets. Supermarkets and specialized FV markets were more stable in areas with low HVI (supermarket: low = 58.3% vs. medium = 37.1% vs. high = 40.0%. Specialized FV market: low = 45.9% vs. medium = 25.9% vs. high = 22.9%), whereas local stores were more stable in areas with a high HVI (low = 23.1%; medium = 45.1%; and high = 51.2%). All supermarkets and local stores in areas with medium and high HVI when closed were not replaced. The closed specialized FV markets showed a lower replacement rate in the low HVI regions (Table 3).

**Table 3:** Food store fluctuations, 2013–2018. Belo Horizonte, Brazil

Stores	Total		Supermarket		Specialized FV markets		Local stores		p-value^a^	
	n	%(95%CI)	n	%(95%IC)	n	%(95%CI)	n	%(95%CI)		
Total										
Stable	168	35.2 (31.0-39.6)	33	44.6 (33.6–56.1)	97	30.7 (25.8–36.0)	38	43.7 (33.6–54.3)	0.076	
Closed	154	32.3 (28.2–36.6)	20	27.0 (18.0-38.3)	110	34.8 (29.7–40.2)	24	27.6 (19.2–38.0)		
*Not replaced*	142	92.2 (86.7–95.5)	20	100.0	99	90.0 (26.4–36.7)	23	95.8 (18.1–36.7)		
*Replaced*	12	7.8 (4.4–13.3)	0	0.0	11	10.0 (1.9–6.2)	1	4.2 (0.1–7.8)		
New	155	32.5 (28.4–36.8)	21	28.4 (19.2–39.7)	109	34.5 (29.4–39.9)	25	28.7 (20.1–39.2)		
Found 2013^b^	322	67.5 (63.1–71.6)	53	71.6 (60.1–80.9)	207	65.5 (60.0-70.6)	62	71.3 (60.7–79.9)	0.426	
Found 2018^c^	323	67.7 (63.4–71.8)	54	73.0 (61.5–82.0)	206	65.2 (59,7-70.3)	63	72.4 (61.9–80.9)	0.255	
**Low HVI**										
Stable	54	45.9 (37.1–54.9)	14	58.3 (37.7–76.4)	39	45.9 (35.5–56.7)	3	23.1 (7.1–5.8)	0.254	
Closed	24	19.7 (13.5–27.8)	2	8.3 (2.0-28.8)	18	21.2 (13.7–31.3)	4	30.8 (11.4–60.5)		
*Not replaced*	19	79.2 (56.8–91.7)	2	100.0	14	77.8 (51.1–92.1)	3	75.0 (16.0-97.9)		
*Replaced*	5	20.8 (8.3–43.2)	0	0.0	4	22.2 (7.8–48.8)	1	25.0 (2.0-84.4)		
New	42	34.3 (26.4–43.4)	8	33.3 (17.2–54.5)	28	32.9 (23.7–43.7)	6	46.1 (21.4–72.9)		
Found 2013^b^	80	65.6 (56.6–73.5)	16	66.7 (44.4–83.3)	57	67.1 (56.2–76.3)	7	53.8 (24.8–80.5)	0.642	
Found 2018^c^	98	80.3 (72.2–86.5)	22	91.7 (69.8–98.1)	67	78.8 (68.6–86.3)	9	69.2 (36.5–89.8)	0.214	
**Medium HVI**										
Stable	80	31.2 (25.8–37.3)	13	37.1 (22.7–54.3)	44	25.9 (19.8–33.0)	23	45.1 (31.9–59.0)	0.097	
Closed	91	35.5 (29.9–41.6)	10	28.6 (15.9–45.8)	67	39.4 (32.3–47.0)	14	27.4 (16.8–41.4)		
*Not replaced*	88	96.7 (90.0-98.9)	10	100.0	64	95.5 (86.7–98.6)	14	100.0		
*Replaced*	3	3.3 (1.0-9.9)	0	0.0	3	4.5 (1.4–13.3)	0	0.0		
New	85	33.2 (27.7–39.2)	12	34.3 (20.4–51.5)	59	34.7 (27.9–42.2)	14	27.4 (16.8–41.4)		
Found 2013^b^	171	66.8 (60.7–72.3)	23	65.7 (47.9–80.0)	111	65.3 (57.8–72.1)	37	72.5 (58.3–83.3)	0.621	
Found 2018^c^	165	64.4 (58.3–70.1)	25	71.4 (53.6–84.4)	103	60.6 (53.0-67.7)	37	72.5 (58.3–83.3)	0.191	
**High HVI**										
Stable	32	32.3 (23.7–42.3)	6	40.0 (18.4–66.3)	14	22.9 (13.9–35.4)	12	52.2 (31.9–71.8)	**0.030**	
Closed	39	39.4 (30.1–49.5)	8	53.3 (28.3–76.8)	25	41.0 (29.2–53.9)	6	26.1 (11.9–48.0)		
*Not replaced*	35	89.7 (74.8–96.3)	8	100.0	21	84.0 (63.0-94.2)	6	100.0		
*Replaced*	4	10.3 (3.7–25.2)	0	0.0	4	16.0 (5.8–37.0)	0	0.0		
New	28	28.3 (20.2–38.1)	1	6.7 (0.1–37.4)	22	36.1 (24.9–49.0)	5	21.7 (9.0-43.6)		
Found 2013^b^	71	71.7 (61.9–79.8)	14	93.3 (58.4–99.3)	39	63.9 (50.9–75.2)	18	78.3 (55.2–91.3)	0.056	
Found 2018^c^	60	60.6 (50.5–69.9)	7	46.7 (21.7–73.4)	36	59.0 (46.0-70.9)	17	73.9 (50.9–88.6)	0.224	

^a^Chi-square: compare food store fluctuations (stable, closed, and new stores) vs. types of stores (supermarket, specialized FV market, and local stores); ^b^Stable + Closed = stores found in 2013; ^c^Stable + New = stores found in 2018. FV = fruits and vegetables; HVI = Health Vulnerability Index; 95% CI: confidence interval. Stables stores: 2013 stores remaining open through 2018; Closed stores: 2013 stores closed in 2018; Replaced stores: 2013 stores that were closed and replacements in 2018; Not replaced stores: 2013 stores that were closed but not replaced in 2018. New stores: 2018 stores that did not exist in 2013.

**Fig. 4:**
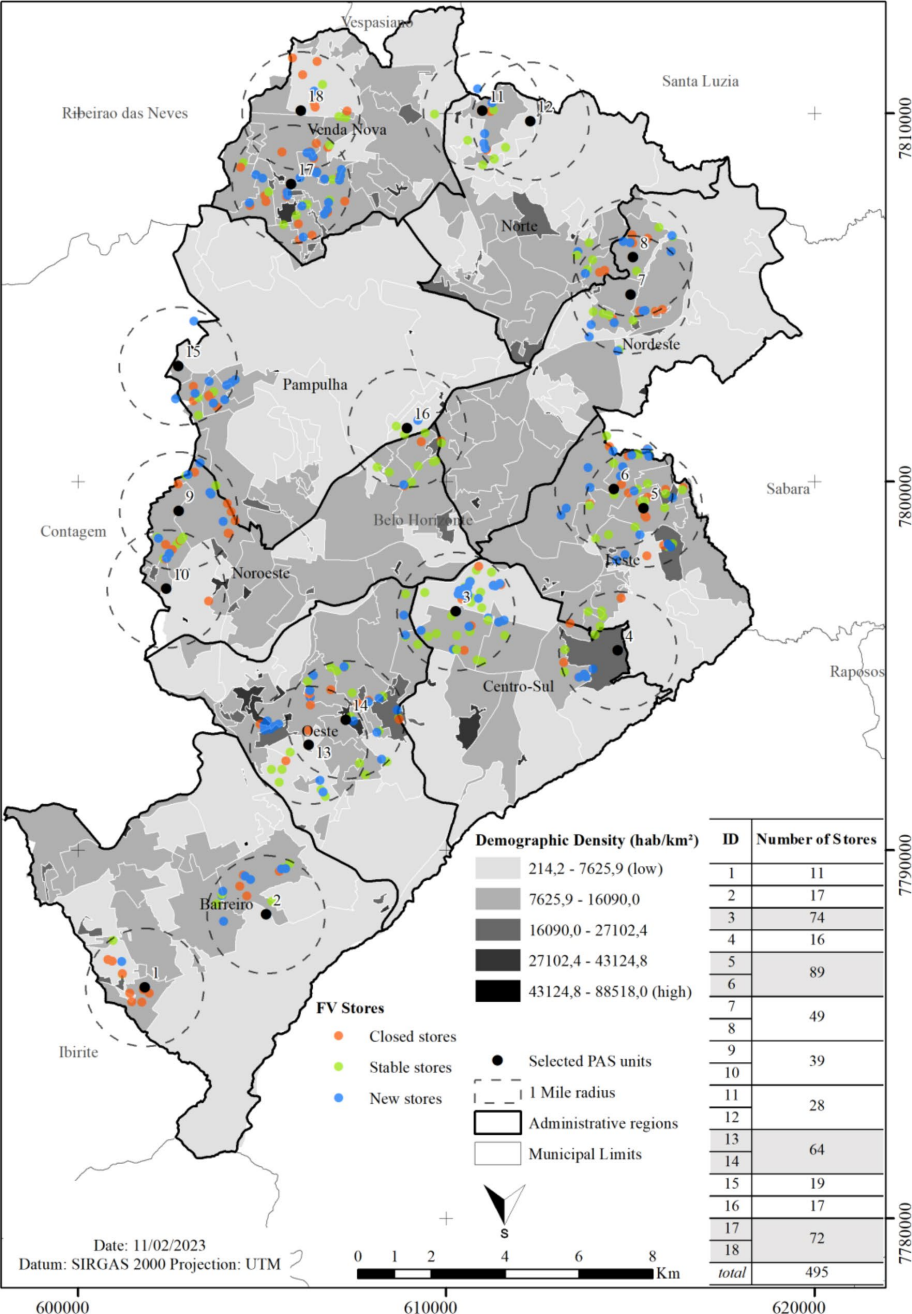
Demographic Density and food stores fluctuation over five years. Belo Horizonte, Brazil, 2013–2018. *Note*: The human development unit (HDU) are units of analysis with relatively homogeneous socioeconomic characteristics, resulting from the aggregation of census tracts with similar characteristics, created for AtlasBR, in a partnership between the United Nations Development Program, and the João Pinheiro Foundation and the Institute of Applied Economic Research. Source: AtlasBR – Atlas Platform for Human Development in Brazil. Available at: www.atlasbrasil.org.br. Accessed: May 5, 2021

A medium demographic density was observed in most of the territories surveyed. In some cases, it can be observed that the distribution of stores followed the demographic concentration, as in radium 1, 2, 5, 6, 7, 8, 10, 14, 15, 16 and 17. However, some cases show a discrepancy population density and concentration of stores, as in radium 3, 4, 13 and 18 (Fig. 4).

## Discussion

In our knowledge, this was the first study to assess changes in the food environment of a Latin American country through an audit. The results showed that the food environment in the PAS areas proved dynamic with the opening and closing of stores; however, the total number of stores, in general, remained stable. The fluctuation of food stores also varied by the vulnerability of the area, being the stores located in less vulnerable areas more stable. The fluctuation differed by type of stores only in high HVI areas; and in areas with medium and high HVI, where the local stores were more present than the supermarkets.

Although the availability of food stores in the area has not changed, the high fluctuation of stores may represent changes in the dynamics of food sales. This finding may reflect the development and population changes of the most vulnerable regions of the city. Data from the last census carried out in 2022, showed a 2.5% reduction in the municipality’s population when compared to data from 2010. However, we still do not have population density data according to the city’s region for this period. It is important to consider that the municipality has limited territory for growth and it is believed that population variation may be a reflection of the aging of the population and migratory flow to areas with lowers costs of living.

In addition, it was found the stability of the stores was greater in the less vulnerable areas. The period of this study (2013–2018) encompassed a political crisis and economic recession in Brazil [17]. Thus, this may also represent how much the low-income populations are more affected by the economic crisis. Businesses in those areas may have fewer resources to withstand economic downturns than those in wealthier areas, and many could not survive for long [18]. Corroborating this hypothesis, another study that verified the change in the retail food environment in the same municipality, but using secondary data, showed changes in the neighborhood’s food environment, over the course of 10 years, were unfavorable for adequate access to healthy foods in lower-income neighborhoods [19].

In our study, only 35.2% of food stores remained stable during the period, and many supermarkets were closed over five years, mainly in high HVI areas. The differences in fluctuation observed according to the vulnerability of the areas were also found in other contexts. In the United Kingdom, the poorest areas showed the greatest changes in food environments from 1990 to 2008 [20]. In a study carried out in Spain, small stores showed little variation between 2013 and 2017, and in areas with worse socioeconomic conditions, the variation was larger [21]. In the USA, from 2009 to 2017, significant declines were observed in the number of convenience stores in lower- and medium-income areas, while an increase was observed in the number of supermarkets in medium-income areas [22]. In the same country, examining 40 years of data, the Framingham Heart Study revealed that access to most food establishment types increased over time, and even though poorer areas had higher access to fast-food restaurants, this disparity, compared to other areas, diminished over time [23].

The lack of replacement of supermarkets and local stores in medium and high HVI areas may impact access to food for the most vulnerable population. This is especially worrying when considering that supermarkets are the main food purchasing establishments in the country and reported by consumers as stores with a wide variety of fresh foods, which is significantly associated with a lower chance of consuming ultra-processed foods (UPF) [24].

On the other hand, the low replacement rate of specialized FV markets in low-HVI areas may suggest increased exposure of the local population to establishments such as supermarkets. This is also worrying since specialized stores are characterized by the supply of fresh food while supermarkets are the main locations for purchasing UPF [3].

Changes in retail food environments in Latin America indicate the growth of supermarkets and convenience stores; and the reduction of traditional retail stores, such as local markets [25]. However, in our study, regardless of the vulnerability of the areas and the period, specialized FV markets were the most common stores. It should be emphasized that the comparison with other contexts needs to be cautious because of differences in commercialization dynamics. Another study carried out in Belo Horizonte showed a lower number of food stores in general in regions with greater economic inequality. Furthermore, in these regions there was less opportunity to have supermarkets [26]. Anyway, food environments with a higher number of specialized FV markets are associated with higher consumption of FV in Latin America [25], which demonstrates the importance of FV stores in promoting adequate and healthy food. In addition, previous research showed that the best access to healthy food in the PAS areas was found in this type of store [7].

In most PAS areas, the number of stores followed demographic density, with reservations. The PAS unit number 3 is located in the central region of the city, which historically has a concentration of services and products on offer and a high circulation of people [27]. This radius also includes part of the hospital area, which receives a large flow of people and from neighboring municipalities. For this reason, even though it is an area with a lower population density than the others, the greater number of food stores are justified by the large circulation of people. Another important observation is the scarcity of stores close to or within the territories of villages and favelas, as an example of radius 4, which has a high demographic density in a large part of the territory, but with few commercial establishments.

PAS units have an average of 190 registered users, which represents an intense flow of people daily using the service. Due to the intense movement in PAS area, it is believed that the stimulus and investment in public equipment for the sale of fresh food in these regions can be an opportunity to enhance the healthy environment. In the city under study, there are important public initiatives to encourage food production, defense, and promotion of food consumption, and subsidized marketing of food (example: stores specializing in FV government-subsidized that provide low-cost FV and open market programs for rural producers). These initiatives need to dialogue with other policies, such as health and urban planning, among others, to promote equity and meet the needs of the most vulnerable communities across sectors [27].

The government should also support food retailers to have more healthy food stores. The public sector can act as a driver of strategies that improve access and availability of healthy food, through the training of retailers, for example. It is necessary to strengthen traders, local producers, and policies that contribute to health promotion. This can have an impact on the health of the population and can stimulate the local economy [28].

This study does have some limitations that should be considered. First, analyzed only the food environment for consumption at home and stores that sell FV. In other contexts, studies analyzing stores that sell food for immediate consumption (including fast food, and restaurants, among others) also highlight greater changes in economically disadvantaged areas [23], which accompanies the results presented here. In any case, further research needs to be conducted to understand the changes in food stores. Second, this study used a 1 mile radium to determine the food environment, considered sensitive for calculating the availability of establishments in the region [15]. The radius of one mile was chosen because it is a walkable distance a usual between adults, and frequently used in other studies [15, 29, 30].

In addition, some areas had a geographic location bordering on other municipalities, where stores were not investigated. There is also a possibility of bias in defining the stability of stores since a new establishment can be opened in place of one that has closed. However, our study maintained the criteria used in other studies to facilitate comparison [18]; and allowed us to show how much stores can fluctuate (close or open). We believe that changing a store (while maintaining the type) can result in changes in availability, variety, price, and quality of food, but this needs to be confirmed in other studies. It is also important to recognize the possibility of informal and street food vendors which can mask the true distribution of the city’s food businesses.

This study highlights the importance of routine food environment surveillance, which helps to identify widening disparities within communities and cities. In addition to being the first study to assess the fluctuation of food stores in the country using audit data, it also shows how this process can occur in different ways, according to the vulnerability of the area or type of store. Carrying out the audit allowed the real characterization of the food environment, which is important considering the low validity of secondary data in the region [7]. Furthermore, this study investigated the food environment in areas with public health promotion services, aligned with public health policies.

Our results can contribute to the development and implementation of planning policies, which are urgently needed, especially in the most vulnerable areas. It is necessary to identify the demand for the implementation and reinforcement of health promotion actions with public food supply policies that favor the construction of healthy food environments. One mechanism would be to invest in the creation of healthy eating environments in regions where there is a health promotion service and a large circulation of people. This investment can be as much to guarantee access to the establishment that sells healthy food, as for the qualification of the merchants. Finally, if the environment changes over time and within a crisis, it is important that policies for food environments be consistent with the demand of the region, and reformulated whenever necessary. In addition, they must include plans of the State and not of Government to guarantee their sustainability over time.

## Conclusion

This study examined changes in the community food environment over time to the vulnerability of the area within PHC in Brazil. Despite the opening and closing of food stores, there is stability food environment. However, there was a change in the dynamics of commercialization in areas of greater vulnerability, characterized by the reduction of supermarkets. More studies are needed to understand how commercial dynamics change can influence the population’s nutrition and health.

Public policies are required to alter the inequities identified, and the policies related to the food environment can be important components of efforts to promote health for people and the environment.

### Electronic supplementary material

Below is the link to the electronic supplementary material.

Supplementary Material 1

## Data Availability

The data used in the research is not publicly accessible. The datasets are available from the corresponding author on reasonable request.

## List of abbreviations

FV: fruits and vegetables.
PAS: *Programa Academia da Saúde, in Portuguese* (Health Academy Program).
PHC: Primary Health Care.
SUS: *Sistema Único de Saúde, in Portuguese (*Unified Health Syste).
NCDs: non-communicable diseases.
CI: confidence interval.
NRLE: National Register of Legal Entities.
HVI: Health Vulnerability Index.
UPF: Ultra-processed foods.
