# Supplementary material for: A longitudinal analysis of the fluctuation of food stores in Belo Horizonte, Minas Gerais, Brazil
Source: BMC Public Health. 2023 Dec 7;23:2454. doi: 10.1186/s12889-023-17350-8 (PMC10704749; doi:10.1186/s12889-023-17350-8)
Supplement: Supplementary file 1 — Supplementary Material 1 [file 12889_2023_17350_MOESM1_ESM.docx]

**Supplementary Material** **1: Selected indicators in each of the dimensions, IVS 2012.**

| **Aspect** | **Indicator** |
| --- | --- |
| *Sanitation* | Percentage of permanent private households with inadequate or absent water supply |
|  | Percentage of permanent private households with inadequate or absent sanitation |
|  | Percentage of permanent private households with inadequate or absent garbage destination |
| *Socioeconomic* | Ratio of residents per household |
|  | Percentage of illiterate people |
|  | Percentage of private households with per capita income up to ½ minimum wage |
|  | Average nominal monthly income of responsible persons (reversed) |
|  | Percentage of black, brown and indigenous people |

SMSA (2012)
